# Cyclic AMP-Responsive Element Modulator *α* Polymorphisms Are Potential Genetic Risks for Systemic Lupus Erythematosus

**DOI:** 10.1155/2015/906086

**Published:** 2015-10-27

**Authors:** Qian Guo, Xuyong Chen, Yan Du, Jianping Guo, Yin Su

**Affiliations:** Department of Rheumatology & Immunology, Peking University People's Hospital, 11 Xizhimen South Street, Beijing 100044, China

## Abstract

To investigate whether the cyclic AMP-responsive element modulator *α* (*CREMα*) polymorphisms are novel susceptibility factors for systemic lupus erythematosus (SLE), four tag SNPs, rs1057108, rs2295415, rs11592925, and rs1148247, were genotyped in 889 SLE cases and 825 healthy controls. Association analyses were performed on whole dataset or clinical/serologic subsets. Association statistics were calculated by age and sex adjusted logistic regression. The G allele frequencies of rs2295415 and rs1057108 were increased in SLE patients, compared with healthy controls (rs2295415: 21.2% versus 17.8%, OR 1.244, *P* = 0.019; rs1057108: 30.8% versus 27.7%, OR 1.165, *P* = 0.049). The haplotype constituted by the two risk alleles “G-G” from rs1057108 and rs2295415 displayed strong association with SLE susceptibility (OR 1.454, *P* = 0.00056). Following stratification by clinical/serologic features, a suggestive association was observed between rs2295415 and anti-Sm antibodies-positive SLE (OR 1.382, *P* = 0.044). Interestingly, a potential protective effect of rs2295415 was observed for SLE patients with renal disorder (OR 0.745, *P* = 0.032). Our data provide first evidence that *CREMα* SNPs rs2295415 and rs1057108 maybe novel genetic susceptibility factors for SLE. SNP rs2295415 appears to confer higher risk to develop anti-Sm antibodies-positive SLE and may play a protective role against lupus nephritis.

## 1. Introduction

Systemic lupus erythematosus (SLE) is a prototypic systemic autoimmune disease which is characterized by complex immunological abnormalities and multiple tissue and organ damage [1]. The etiology of SLE has not been fully understood, but it is widely accepted that the interaction between genetic and environmental factors contributes to SLE pathogenesis [2, 3]. Previous studies have shown that genetic factors are the major determinants leading to the susceptibility of SLE, and to date more than 40 genetic loci have been proven to be associated with SLE [4, 5].

Cyclic AMP-responsive element modulator (CREM) proteins are members of the leucine zipper protein superfamily of nuclear transcription factors. They are key regulators of cAMP-mediated signal transduction. Human CREM proteins are encoded by the CREM gene, which comprised over 20 alternatively spliced isoforms, including CREM*α*. CREM*α* is a widely expressed transcriptional repressor that is important in regulation of T cell immune response [6, 7]. Evidence has shown that CREM*α* was overexpressed in T cells from patients with SLE [8, 9]. Previous study reported that CREM*α* mRNA expression was increased in T cells from SLE patients, though it did not correlate with clinical features, disease activity, or therapeutic effects; patients with high doses of corticosteroids had a trend to possess low CREM*α* mRNA levels [10]. Despite a number of immunological studies of CREM*α* in SLE, however, to date, there is no any report on genetic susceptibility of* CREMα* in SLE. This study therefore aimed to investigate whether the genetic variant(s) in human* CREMα* is associated with SLE susceptibility and to evaluate whether* CREMα* polymorphism(s) is associated with any clinical/serologic features in SLE. To the best of our knowledge, this is the first time we report that two tag single-nucleotide polymorphisms (SNPs) rs2295415 and rs1057108 from* CREMα* are novel susceptibility factors for SLE. SNP rs2295415 may confer increased risk to developing of anti-Smith (Sm) antibodies-positive SLE and may have a protective role in patients with renal disorder.

## 2. Materials and Methods

### 2.1. Study Subjects

A total of 889 patients with SLE and 825 nonrelated healthy controls were enrolled in the study. The patients with SLE were recruited from the Department of Rheumatology and Immunology from Peking University People's Hospital and People's Hospital of Xinjiang Province. The healthy controls were recruited from Health Care Centers of Peking University People's Hospital. In the Health Care Centers, thousands of residents come for annual regular physical examination from the local geographical areas. The healthy controls were selected from these residents without any disease records. All patients and healthy controls are Han Chinese.

The patients with SLE fulfilled 1997 revised American College of Rheumatology (ACR) classification criteria for SLE [11] and were selected without developing other rheumatic diseases. The patients with renal disorder were defined by the following criteria: (a) persistent proteinuria greater than 0.5 g per day or greater than +++ if quantization is not performed or (b) cellular casts which may be red cell, hemoglobin, granular, tubular, or mixed. Anti-double-stranded DNA (dsDNA) antibodies were measured with enzyme-linked immunosorbent assay (ELISA, Kexin Biotechnology Ltd., Shanghai, China). Values >100 IU/mL were assessed as positive. Anti-Sm antibodies were determined by an immunoblot method from Euroimmun (Lübeck, Germany), and results were reported as positive or negative in relation to reference sera. The data for the two autoantibodies were available for all the in-patients and part of out-patients due to the incomplete records in electronic system in out-patient department.

The characteristics of patients and healthy controls were detailed in Table 1. The study was approved by Medical Ethics Committee in Peking University People's Hospital and the informed consents were obtained from all participants.

### 2.2. TagSNP Selection and Genotyping

**Figure 1:**
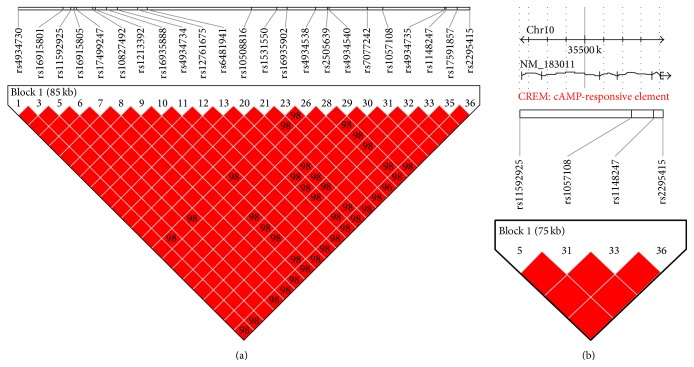
(a) Thirty-six* CREMα* SNPs were shown with linkage disequilibrium (LD); the intensity of LD is reflected in the color and digital value of each box. (b) The LD structure and location of four tagSNPs. Red represents strong linkage; white represents no linkage. Digital value in each box represents the *D*′ values ×100 for linkage disequilibrium between the two corresponding SNPs; the maximum *D*′ value is 1, which indicates complete linkage.

The tagSNPs were selected from the CHB panel (Han Chinese in Beijing) of HapMap project (the Phase II database, http://hapmap.ncbi.nlm.nih.gov/). The criterion for tagging was set at *D*′ > 0.8 and minor allele frequency (MAF) > 0.05. By using Haploview 4.2 software, a total of 4 tagSNPs were identified: rs1057108, rs11592925, rs114824, and rs2295415 (Figures 1(a) and 1(b)).

Genotyping of SNPs rs1148247 and rs2295415 was performed using predesigned TaqMan SNP Genotyping Assays (C_8876200_10 and C_16189959_10, resp., Applied Biosystems, Foster City, California). Allelic discrimination was performed in ABI 7300 Real-Time PCR system (Applied Biosystems). The genotyping successful rate was 99.4%.

SNPs rs1057108 and rs11592925 were genotyped using Sequenom MassArray platform with primers and probes (Supplementary Table S1 in Supplementary Material available online at http://dx.doi.org/10.1155/2015/906086). Briefly, DNA from case and control subjects was randomly assigned to the 96-well plates, and genotyping was performed, blind to the status of the samples. Genotyping was repeated in 5% of samples for validation and quality control. The genotype data had an error rate less than 0.1%.

### 2.3. Power Analysis

The power analyses were performed retrospectively for the available samples (cases and controls), according to MAF for each SNP, type I error *P* of 0.05, and an odds ratio (OR) of 1.40. The Power and Sample-size (PS) software (version 3.0.14) was used for the power calculation (available at http://biostat.mc.vanderbilt.edu/wiki/Main/PowerSampleSize).

### 2.4. Statistical Analysis

Hardy-Weinberg equilibrium was assessed for each polymorphism, using Pearson's goodness-of-fit chi-square test. The chi-square tests with continuity correction were performed for the comparisons of allelic frequency differences and haplotypes between patients and controls. The linkage disequilibrium (LD) and haplotype were calculated using online software SHEsis (http://analysis.bio-x.cn/myAnalysis.php). ORs and 95% confidence intervals (CI) for alternative genetic model analysis were calculated using logistic regression, adjusting for age and sex. All statistical analyses were conducted using SPSS 13.0 (SPSS Inc., Chicago, IL, USA). *P* values less than 0.05 were considered statistically significant.

## 3. Results

SNPs rs2295415, rs1057108, rs11592925, and rs1148247 were in Hardy-Weinberg equilibrium in both patients and controls (*P* > 0.05, Supplementary Table S2), illustrating that the subjects were collected from a random mating population. In control group, the allele frequencies of the four SNPs were similar to the data from HapMap CHB. The study has a statistical power of greater than 0.807 to detect the significant effect between the tagSNPs and SLE, except for rs11592925 (study power = 0.609).

### 3.1. Association of CREM*α* Polymorphisms with Susceptibility to SLE at Allele and Genotype Level

We first sought to determine whether there was an association between* CREMα* polymorphisms and SLE susceptibility. As shown in Table 2, at allele level, we observed significant higher minor allele frequencies of rs2295415 and rs1057108 in patients with SLE, compared with healthy controls (rs2295415: 21.2% versus 17.8%, OR 1.244, 95% CI 1.040–1.487, *P* = 0.019; rs1057108: 30.8% versus 27.7%, OR 1.165, 95% CI 1.003–1.152, *P* = 0.049). At genotype level, SNP rs2295415 displayed a significant association with SLE susceptibility (codominant model: OR = 1.241, 95% CI 1.009–1.527, *P* = 0.041, Table 3). A suggestive association was also found between rs1057108 and SLE (codominant model: OR = 1.185, 95% CI 0.999–1.405, *P* = 0.052). No association was observed for rs11592925 and rs1148247 in SLE susceptibility.

### 3.2. Association of CREM*α* Polymorphisms with Susceptibility to SLE at Haplotype Level

Haplotypes were constructed using the two susceptible SNPs rs1057108 and rs2295415 (*D*′ = 0.82). As shown in Table 4, a total of 4 haplotypes were identified. Haplotype T-A was the major composition (63.8% versus 65.3%). The haplotype constituted by the two risk alleles “G-G” displayed strong risk effects contributed to SLE susceptibility (OR 1.454, 95%CI 1.175–1.799, and *P* = 0.00056). In contrast, other haplotypes showed no association.

### 3.3. Association of CREM*α* Polymorphisms with SLE Subphenotypes

Next, we sought to determine whether there was any association between the risk SNP rs2295415 and any specific clinical/serologic manifestations in SLE. Following stratification by clinical/serologic features, we found higher frequencies of rs2295415 G allele in anti-Sm antibodies-positive patients, compared with healthy controls (22.8% versus 17.8%, OR 1.382, 95% CI 1.015–1.883, and *P* = 0.044, Table 5). A similar result was also observed for anti-dsDNA antibodies-positive patients, though they did not reach the statistical significance (20.3% versus 17.8%, OR 1.177, 95% CI 0.937–1.478, and *P* = 0.173). Interestingly, a potential protective effect of rs2295415 was observed for SLE patients with renal disorder (OR 0.745, 95% CI 0.570–0.973, and *P* = 0.032).

## 4. Discussion

Although several genetic studies have showed that the SNPs rs2295415 and rs1057108 were significantly associated with ulcerative colitis and Crohn's disease [12–14] and the immunological studies have indicated that CREM*α* is implicated in the pathogenesis of SLE, to data, there are no genetic studies of* CREMα* in SLE susceptibility. Therefore, we undertook the current study to investigate whether human* CREMα* polymorphisms play a role in SLE susceptibility. Our results indicated that* CREMα* SNPs rs2295415 and rs1057108 may be novel genetic risk factors contributing to SLE susceptibility in Han population. SNP rs2295415 G allele conferred a potential risk to develop anti-Sm antibodies-positive SLE and appeared to have a protective role in patients with renal disorder.

The precise function of CREM*α* is unknown. However, the existing data suggest that CREM*α* can specifically bind to the IL-2 promoter, leading to a repression of IL-2 transcription [15, 16]. CREM*α* overexpression in SLE T cells resulted in enhanced binding of CREM*α* to IL-2 promoter and reduced IL-2 expression [17]. It is clear that IL-2 is a critical cytokine produced by T cells upon activation and is important for the generation of T regulatory cells and activation-induced cell death [18]. In SLE patients, T cells display decreased capacity to produce IL-2 [19]. Impaired IL-2 expression resulted in decreased generation of regulatory T lymphocytes [20] and defect of activation-induced cell death [21, 22]. These findings suggest an important role of CREM*α* in regulation of IL-2 and in the pathogenesis of SLE.

The mechanism(s) underlying this genetic association remains elusive. It may be explained by the fact that rs2295415 is located in 3′ UTR of* CREMα*, the region implicated in the regulation of gene expression. A number of disease-associated polymorphisms have been mapped in the 3′ UTR of protein-coding genes [23, 24]. The significant association of SNP rs2295415 with SLE susceptibility and subphenotypes may be due to its effect on* CREMα* gene expression and/or its LD with a functional variant residing in a neighboring gene(s). However, in present study, we also showed that another tagSNP rs1057108 was also significantly associated with SLE. Thus, we currently cannot exclude that rs1057108 might be independent genetic risk contributing to SLE susceptibility, though the SNP may be a nonfunctional variant since it is resided in the intronic region and has not been annotated as any promoter, enhancer, repressor, or distant regulatory element (NCBI http://www.ncbi.nlm.nih.gov/ MAF Source: 1000 Genomes). The functional consequences of the SLE-risk SNPs need to be elucidated in the future studies.

Identification of genetic risk factors responsible for disease subsets/subphenotypes is important for the understanding of disease pathogenesis. It has been shown that certain genetic association was restricted to clinical and autoantibody subsets in SLE [25]. In present study, we found a suggestive association between rs2295415 G allele and anti-Sm antibodies-positive SLE and a trend association between rs2295415 G allele and anti-dsDNA antibodies-positive SLE. Another interesting finding of our work is the potential protective effect of rs2295415 G allele in patients with renal disorder. However, in present work, the case numbers were relatively small after stratification for clinical/serologic subsets. As a result of the modest sample size, there may be a risk that observed findings are due to chance. Additional studies with larger sample sizes are desired to confirm our findings.

In conclusion, our study provides the first evidence that* CREMα* SNPs rs2295415 and rs1057108 may be novel genetic susceptibility factors for SLE, especially at haplotype level. SNP rs2295415 appears to confer higher risk to develop autoantibody-positive diseases, such as anti-Sm antibodies-positive and anti-dsDNA antibodies-positive SLE, and may play a protective role against lupus nephritis.

## Supplementary Material

Table S1: Primer sequences for SNP genotyping using Sequenom MassArray method.
Table S2: Hardy-Weinberg equilibrium test in SLE cases and healthy controls.

## Figures and Tables

**Table 1:** Demographic and clinical characteristics of subjects.

Characteristics	SLE cases (*n* = 889)	Controls (*n* = 825)
Female (%)	90.0	88.5
Age (mean ± SD years)	36 ± 13	43 ± 9
Age of onset (mean ± SD years)	29.0 ± 0.6	—
Disease duration (mean ± SD years)	5.4 ± 0.3	—
Clinical manifestations (%)		
Rash (*n* = 392)	44.1	—
Arthritis (*n* = 386)	43.4	—
Renal disorder (*n* = 315)	35.4	—
Autoantibody positivity (%)		
Anti-dsDNA positivity (*n* = 364)	41.0	—
Anti-Sm positivity (*n* = 151)	17.0	—

SLE: systemic lupus erythematosus; anti-dsDNA: anti-double-stranded DNA antibody; anti-Sm: anti-Smith antibody; SD: standard deviation.

**Table 2:** Allele analysis of* CREMα* tagSNPs in SLE association.

SNPs	Allele	Allelic frequencies (cases versus controls)	MAF (cases versus controls)	OR (95% CI)	*P* values
rs1057108	G/T	542/1216, 444/1160	0.308, 0.277	1.165 (1.003–1.152)	**0.049**
rs2295415	G/A	343/1275, 265/1225	0.212, 0.178	1.244 (1.040–1.478)	**0.019**
rs11592925	T/C	169/1591, 159/1447	0.096, 0.099	0.967 (0.770–1.214)	0.772
rs1148247	A/G	574/1054, 536/948	0.353, 0.361	0.963 (0.832-1.116)	0.626

*CREMα*: cyclic AMP-responsive element modulator *α*; SNPs: single-nucleotide polymorphisms; MAF: minor allele frequency; OR: odds ratio; CI: confidence interval.

**Table 3:** Genotype analysis of *CREMα* tagSNPs in SLE association, adjusting for sex and age.

SNPs	Genotype	Cases (%)	Controls (%)	Codominant		Dominant		Recessive	
				OR (95% CI)	*P* values	OR (95% CI)	*P* values	OR (95% CI)	*P* values
rs1057108	TT	422 (48)	417 (52)	1.185 (0.999–1.405)	0.052	1.200 (0.966–1.491)	0.100	1.363 (0.916–2.028)	0.127
	TG	372 (42)	326 (41)						
	GG	85 (10)	59 (7)						

rs2295415	AA	498 (62)	497 (67)	**1.241 **(1.009–1.527)	**0.041**	1.222 (0.964–1.549)	0.098	1.873 (0.967–3.625)	0.063
	AG	279 (34)	231 (31)						
	GG	32 (5)	17 (2)						

rs11592925	CC	719 (82)	654 (82)	1.049 (0.813–1.354)	0.712	1.055 (0.799–1.394)	0.704	1.046 (0.369–2.964)	0.933
	CT	153 (17)	139 (17)						
	TT	8 (1)	10 (1)						

rs1148247	GG	349 (43)	307 (41)	0.934 (0.794–1.10)	0.416	0.916 (0.729–1.150)	0.447	0.913 (0.657–1.629)	0.588
	GA	356 (44)	334 (45)						
	AA	109 (13)	101 (14)						

*CREMα*: cyclic AMP-responsive element modulator *α*; SNPs: single-nucleotide polymorphisms; SLE: systemic lupus erythematosus; OR: odds ratio; CI: confidence interval.

**Table 4:** Haplotype analysis between rs1057108 and rs2295415 in SLE association.

Haplotype	Cases (%)	Controls (%)	*χ* ^2^	*P* values	OR (95% CI)
G-G	244 (15.3)	160 (11.0)	11.927	**0.00056**	**1.454 (1.175~1.799)**
G-A	242 (15.1)	245 (16.8)	1.637	0.207	0.881 (0.726~1.070)
T-A	1021 (63.8)	949 (65.3)	0.770	0.380	0.936 (0.807~1.085)
T-G	93 (5.8)	100 (6.9)	1.351	0.245	0.841 (0.628~1.126)

SLE: systemic lupus erythematosus; OR: odds ratio; CI: confidence interval.

**Table 5:** Association analyses between rs2295415 and subphenotypes in SLE.

Subjects	MAF (%)	OR (95% CI)	*P* values
Controls (*n* = 745)	17.8		
Subphenotypes (positive)			
Rash (*n* = 366)	19.7	1.132 (0.904–1.418)	0.295
Arthritis (*n* = 357)	18.2	1.029 (0.816–1.298)	0.813
Renal disorder (*n* = 299)	13.9	**0.745** (0.570–0.973)	**0.032**
Anti-dsDNA (*n* = 345)	20.3	1.177 (0.937–1.478)	0.173
Anti-Sm (*n* = 139)	22.8	**1.382** (1.015–1.883)	**0.044**

SLE: systemic lupus erythematosus; anti-dsDNA: anti-double-stranded DNA antibody; anti-Sm: anti-Smith antibody; OR: odds ratio; CI: confidence interval.
