# Supplementary material for: Cyclic AMP-Responsive Element Modulator α Polymorphisms Are Potential Genetic Risks for Systemic Lupus Erythematosus
Source: J Immunol Res. 2015 Oct 27;2015:906086. doi: 10.1155/2015/906086 (PMC4639656; doi:10.1155/2015/906086)
Supplement: Supplementary file 1 — Table S1: Primer sequences for SNP genotyping using Sequenom MassArray method. Table S2: Hardy-Weinberg equilibrium test in SLE cases and healthy controls. [file 906086.f1.pdf]

Supplementary Tables

Table S1 Primer sequences for SNP genotyping using Sequenom MassArray method

SNP	Forward primer	Reverse primer	Extension primer
rs1057108	ACGTTGGATGCTCCAAGCTGCCTTTTAGAC	ACGTTGGATGCATGACGTGTTTCCCTCTTC	GGATTTAACATTTCTTTTGAAGTGG
rs11592925	ACGTTGGATGACAGACATATGGTGTGTGCC	ACGTTGGATGGAATACATTCCCTACTCCCC	CGGCAGGAAAACATCTTGAA

SNPs: single-nucleotide polymorphisms

Table S2 Hardy- Weinberg equilibrium test in SLE cases and healthy controls

**SNPs**	**SLE cases**				**Controls**			
	AA	Aa	aa	*p*HW	AA	Aa	aa	*p*HW
rs2295415	498	279	32	0.36	497	231	17	0.10
rs1057108	422	372	85	0.81	417	326	59	0.67
rs11592925	719	153	8	0.96	654	139	10	0.40
rs1148247	349	356	109	0.23	307	334	101	0.50

SNPs: single-nucleotide polymorphisms; SLE: systemic lupus erythematosus; *p*HW: *p* values for Hardy- Weinberg equilibrium test; AA, Aa, aa: three different genotypes
